# NF-κB in Thyroid Cancer: An Update

**DOI:** 10.3390/ijms252111464

**Published:** 2024-10-25

**Authors:** Elvira Crescenzi, Antonio Leonardi, Francesco Pacifico

**Affiliations:** 1 Istituto per l’Endocrinologia e l’Oncologia Sperimentale, Consiglio Nazionale delle Ricerche (CNR), Via S. Pansini, 5, 80131 Naples, Italy; e.crescenzi@ieos.cnr.it; 2 Dipartimento di Medicina Molecolare e Biotecnologie Mediche, University of Naples Federico II, Via S. Pansini, 5, 80131 Naples, Italy; leonardi@unina.it

**Keywords:** NF-κB, thyroid cancer, target genes

## Abstract

The dysregulated NF-κB basal activity is a common feature of human thyroid carcinomas, especially in poorly differentiated or undifferentiated forms that, even if rare, are often resistant to standard therapies, and, therefore, are uncurable. Despite the molecular mechanisms leading to NF-κB activation in thyroid cancer being only partially understood, during the last few years, it has become clear that NF-κB contributes in different ways to the oncogenic potential of thyroid neoplastic cells. Indeed, it enhances their proliferation and viability, promotes their migration to and colonization of distant organs, and fuels their microenvironment. In addition, NF-κB signaling plays an important role in cancer stem cells from more aggressive thyroid carcinomas. Interfering with the different upstream and/or downstream pathways that drive NF-κB activity in thyroid neoplastic cells is an attractive strategy for the development of novel therapeutic drugs capable of overcoming the therapy resistance of advanced thyroid carcinomas. This review focuses on the recent findings about the key functions of NF-κB in thyroid cancer and discusses the potential implications of targeting NF-κB in advanced thyroid carcinomas.

## 1. Thyroid Cancer: An Overview

In the context of endocrine tumors, thyroid carcinoma is the most common malignancy. Its incidence is increasing globally, mainly due to detection by ultrasound imaging and fine-needle aspiration biopsies during screening campaigns. Tumors arising from epithelial follicular cells are represented by papillary (PTC), follicular (FTC), poorly differentiated (PDTC), and anaplastic (ATC) thyroid carcinomas, while those arising from parafollicular C cells are defined as medullary thyroid carcinomas (MTCs). PTCs and FTCs are the well-differentiated (DTC) forms, account for the majority of thyroid cancers, and have an excellent prognosis. PDTCs and ATCs, both originating from DTCs as a result of the accumulation of multiple genetic mutations, constitute the undifferentiated types. They are uncommon but, unfortunately, lethal. MTCs are much less frequent than PTCs and FTCs, but their prognosis is usually less favorable than well-differentiated thyroid cancers [1]. Surgery and, eventually, radioiodine therapy are the gold standard regimens in a large part of the DTC treatments. Radioiodine resistance could develop in a small percentage of PTC and FTC patients, giving rise to local relapse or metastatic disease. In these cases, taking advantage of the oncogene mutational signature of thyroid cancer cells, systemic targeted therapy is provided. PDTCs and ATCs are more difficult to treat because of the high rate of local recurrence and the evidence of distant metastases at the time of diagnosis. In the past, surgery was followed by radio and chemotherapy administration, but, given their very low efficacy, during recent years, palliative care of relapse and/or metastatic disease mainly relies on targeted therapy [2,3].

It is now accepted that DTCs may arise from preexisting benign tumors of follicular cells showing specific morphological features and genetic alterations, but they can also arise de novo. Undifferentiated malignancies usually originate from preexisting DTCs, but they too may develop de novo [4,5,6]. Thyroid carcinogenesis is driven by mutations or rearrangements of specific genes responsible for thyroid cancer cell proliferation and survival, as well as thyroid cancer progression via different signaling pathways involving the activation of MAPK/PI3K/AKT cascades. The majority of these alterations are mutually exclusive activating mutations in *BRAF* or *RAS* oncogenes and rearrangements of *RET*, *ALK*, and *NTRK* genes. A small fraction is represented by loss-of-function mutations of tumor suppressor genes, such as *PTEN*, *PPARγ*, and *TP53* [7].

PTCs are prevalently characterized by *BRAF* mutations or *RET* rearrangements. The most common mutation occurring in the *BRAF* oncogene is represented by the substitution at codon 600 of valine (V) with glutamic acid (E), which generates the constitutively active BRAFV600E kinase [8]. This BRAF mutant, besides promoting cancer initiation through the enhancement of thyroid carcinoma cell growth rate and survival, drives cancer progression through the inhibition of sodium-iodide symporter expression that, in turn, determines iodine unresponsiveness [9]. In fact, BRAFV600E has also been found in the majority of PDTCs and ATCs, and which evolved from the progressive dedifferentiation of prior PTCs [10]. The other mutually exclusive alteration frequently encountered in PTCs is the rearrangement of the gene encoding for the tyrosine kinase receptor RET [11]. The most common rearrangements arise from the fusion between the amino-terminal portion of different proteins with the RET tyrosine kinase domain that leads to the constitutive activation of the receptor. RET/PTC1 (a result of RET and CCDC6/H4 fusion) and RET/PTC3 (a result of RET and NCOA4/RFG/ELE1 fusion) are the most frequent rearrangements found in PTCs affecting younger patients and those with a history of radiation exposure (particularly after the Chernobyl accident). Altered RET function is also common in MTCs, where point mutations affecting RET extracellular or intracellular domains are mainly detected [10].

On the other hand, mutations in the *RAS* oncogene or *PPARγ* tumor suppressor gene frequently occur in FTCs. RAS mutated proteins (KRAS, HRAS, and NRAS) promote the uncontrolled growth of thyroid carcinoma cells, thereby contributing mostly to cancer initiation. Even so, FTCs displaying NRAS protein (one of the most common RAS mutated forms in thyroid cancer) are associated with a higher risk of metastasis. KRAS, HRAS, and NRAS are also detected in PDTCs and ATCs and, to a lesser extent, in the aggressive follicular variant of PTC [12]. Some cases of FTC show altered expression of the *PPARγ/PAX8* gene that arises from the fusion between the thyroid-specific transcription factor PAX8 and the adipocyte nuclear receptor PPARγ. PPARγ/PAX8 is effectively considered an oncoprotein since it can serve as a PPARγ dominant-negative and/or as a PPARγ-like transcription factor. In addition, it can activate or repress Pax8-dependent genes [13].

**Figure 1:**
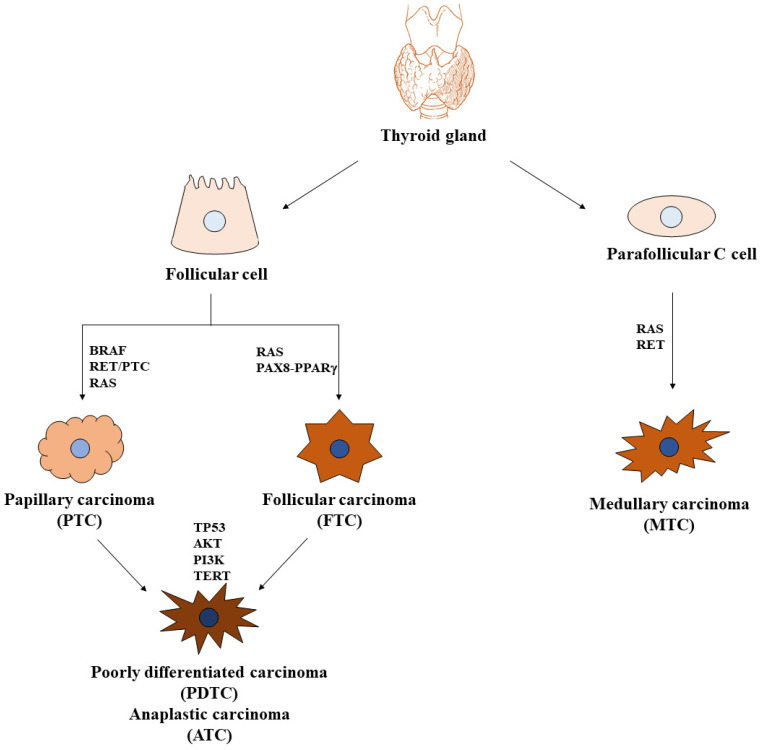
Thyroid carcinomas and key signaling pathways. Two main cell populations are found in the normal thyroid gland: follicular cells, structurally organized to form thyroid follicles, where they secrete thyroglobulin, and parafollicular C cells, which synthesize, store, and secrete calcitonin. Tumors derived from follicular cells are papillary and follicular carcinomas (PTCs and FTCs, respectively), also defined as differentiated carcinomas (DTCs), that could evolve into poorly differentiated and anaplastic carcinomas (PDTCs and ATCs, respectively). Tumors derived from parafollicular C cells are medullary carcinomas (MTCs). The most important mutated genes involved in thyroid carcinogenesis are indicated.

In line with the evidence that the onset of a number of PDTCs and ATCs is the result of the progressive evolution of preexisting DTCs toward a more malignant phenotype (during histological analysis of DTC specimens, it is common to discover small areas of undifferentiated cancer as part of more aggressive DTC), many of the genetic alterations encountered in DTCs are also found in advanced thyroid cancers. Additionally, PDTCs and ATCs are characterized by mutations in the *PTEN* tumor suppressor gene, with the consequent constitutive activation of PI3K/AKT signaling pathways, as well in *TERT* and *TP53* genes, whose dysregulation leads to tumor heterogeneity, dedifferentiation, and progression [14] (Figure 1).

A well-established key player in thyroid cancer is NF-κB. Since the first report of its involvement in the neoplastic transformation of thyroid cells [15], increasingly convincing evidence has accumulated over the years in support of NF-κB’s ability to interfere with many aspects of thyroid carcinoma cell biology [16,17,18,19,20]. The pivotal role of NF-κB is particularly prominent in advanced thyroid tumors, given that its constitutive activity is strongly detectable in human specimens from PDTC and ATC tissues [21,22] and that its signaling is required for ATC cancer stem cells self-renewal and tumorigenic activity [23].

## 2. NF-κB

### 2.1. Physiology

**Figure 2:**
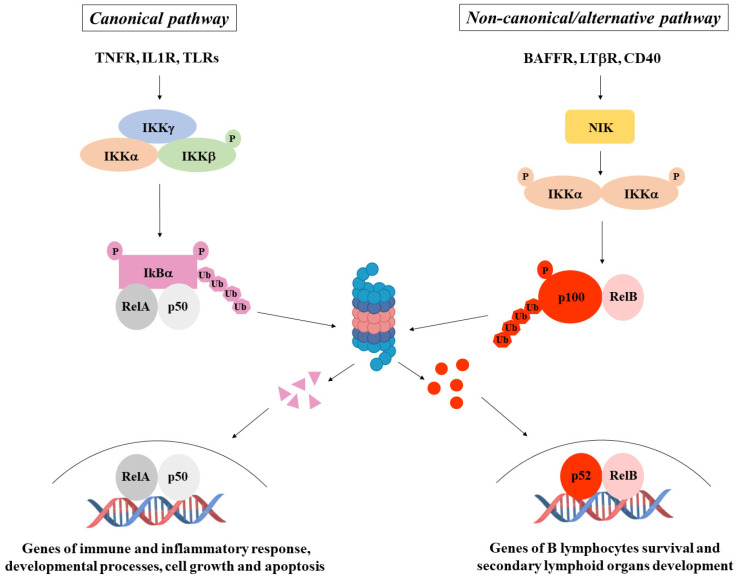
NF-κB pathways. NF-κB activation occurs through a canonical pathway (on the left), generally triggered by immune and inflammatory stimuli, and a non-canonical/alternative pathway (on the right), mainly induced by genes regulating B lymphocyte survival and secondary lymphoid organ development.

NF-κB is a transcription factor family consisting of five members: NF-κB1 (p105/p50), NF-κB2 (p100/p52), RelA (p65), RelB, and c-Rel. They form homo- and hetero-dimers and are able to signal through two major pathways: the canonical pathway, achieved by dimers composed of RelA, c-Rel, and p50, among which RelA/p50 is the most common, and the alternative (also called non-canonical) pathway, whose actors are mainly NF-κB2/RelB dimers. The former is tightly regulated by the inhibitor of NF-κB (IκB), which holds the dimers in the cytoplasm in inactive status to avoid their nuclear translocation. In response to different extracellular or intracellular stimuli, IκB is phosphorylated, ubiquitinated, and degraded by the proteasome to allow the NF-κB dimers to move to the nucleus, where they trigger transcription of genes involved in the regulation of inflammatory and immune response, cell proliferation, and survival. The latter is activated by members of the TNF receptor superfamily that induce NF-κB2 phosphorylation, ubiquitination, and partial degradation in order to generate the mature form p52. In this way, RelB/p52 dimers can form and translocate to the nucleus to activate the transcription of genes involved in secondary lymphoid organ development and lymphocyte survival. Signals activating the canonical pathway converge on a high molecular weight complex—the IκB Kinase (IKK) complex, made up of two catalytic subunits (IKKα and IKKβ) and one regulatory subunit (IKKγ)—while signals activating the alternative pathway converge on a protein kinase called NIK. Canonical pathway activators mediate the phosphorylation of IKKβ, which directly phosphorylates IκB to induce its degradation and allows RelA/p50 dimers to enter the nucleus. Importantly, the regulatory IKKγ subunit, which does not have enzymatic activity but connects upstream signals from inducers to catalytic IKKs, plays a crucial role in that its absence does not allow the correct activation of the IKK complex. In the alternative pathway, IKKα, following phosphorylation mediated by NIK, phosphorylates in turn NF-κB2, promoting the degradation of p100 to p52, thus allowing p52/RelB dimers nuclear translocation (Figure 2).

NF-κB activity is critical for the regulation of many physiologic and pathologic processes. It is essential for inflammatory and immune responses, peripheral lymphoid organ development, immune cell maturation and activation, cell metabolism, and homeostasis. However, when aberrantly activated, NF-κB is involved in different human pathologies, including inflammatory diseases, autoimmune disorders, viral infections, infectious shock, cardiovascular and neurologic diseases, metabolic disorders, and cancer [24].

### 2.2. Cancer

NF-κB plays key functions in neoplastic cells: its dysregulated activity is a common feature of many human tumors, either of solid or haematologic origin. Unlike in normal cells, where the negative feedback control of NF-κB shuttling between cytoplasm and nucleus allows the tight regulation of its activity, in cancer cells, this mechanism is impaired so that NF-κB is constitutively located in the nucleus, where it actively and repeatedly induces transcription of many genes involved in different aspects of tumor biology. The resulting proteins include growth factors, cyclins, antiapoptotic factors, proangiogenic factors, adhesion and epithelial mesenchymal transition (EMT) molecules, cytokines, chemokines, and metabolic enzymes. Thus, NF-κB takes part in different ways in all the main activities that characterize neoplastic cells: it contributes to keeping up the proliferative rate of cancer cells and protects them from death, favoring the onset and the development of tumors [25,26]; it promotes EMT and angiogenesis leading to the local invasion of cancer cells and the formation of distant metastases [27,28]; and it shapes a tumor microenvironment that supports cancer growth by enhancing the protumoral functions of stromal, inflammatory, and immune cells and by suppressing the antineoplastic response of the immune system [29]. In addition, it has been demonstrated that in some types of chronic inflammatory diseases (i.e., *Helicobacter pylori*-dependent gastritis, inflammatory bowel disease, chronic hepatitis), NF-κB is able to establish a network between stromal, immune, and epithelial cells that mutually sustains its constitutive activity in all cell types, enhancing the risk of neoplastic transformation of normal tissue cells. Some gastric carcinomas, colon-carcinomas, and hepato-carcinomas arise from the malignant evolution of NF-κB-driven chronic inflammation [29]. Importantly, increasing evidence indicates that NF-κB is crucial for cancer stem cell generation and function. Cancer stem cells from different tumors show IL-1- and TNF receptor superfamily (TNFSF)-dependent NF-κB activation that determines the transcription of EMT genes, such as *NANOG*, *SNAIL*, *SLUG*, *ZEB1*, *ZEB2,* and *TWIST*, as well *MMP-2* and *MMP-9*. The induction of this transcriptional program allows cancer stem cells to acquire the mesenchymal phenotype with invasive properties, thus fostering cancer aggressiveness. NF-κB also contributes to cancer stem cell self-renewal through the ability of IL-1 to sustain its constitutive activity in an autocrine fashion [30]. Finally, NF-κB also plays a prominent role in cancer-associated cellular senescence induced by intrinsic or therapeutic stresses. Its activity, in fact, results in the establishment of a protumorigenic program of senescent cells via the upregulation of specific factors of the senescence-associated secretory phenotype (SASP), including proinflammatory cytokines (IL-1α, IL-1β, IL-6, and IL-8), chemokines (CCL-2, CCL-5, and CXCL-1), growth factors (HGF, EGF, and TGFα), and matrix-remodeling enzymes (MMP-1 and MMP-3) [31]. These factors work as messengers able to connect senescent cells with their surrounding cells, among which are stromal, immune, and neoplastic cells. The resulting network in the tumor microenvironment not only promotes cancer progression, enhancing tumor invasion and preventing antitumoral immunity, but also increases the risk of relapse because some SASP components may allow senescent cells to evade senescence and acquire stemness properties [31].

Several mechanisms, best characterized in haematologic tumors, concur to aberrant NF-κB activation in malignancies: mutations occurring in core NF-κB pathway proteins and upstream activators, autocrine stimulation, epigenetic and genomic effects, and oncogenic viruses [32]. For example, mutations in different activators of both canonical and alternative NF-κB pathways have been found in about 20% of multiple myeloma patients [33,34] and in about 40% of diffuse large B-cell lymphoma patients [35,36]. Interestingly, NF-κB persistent activation in cancer cells could also be triggered by dysregulated signaling from PI3K/AKT, MAPK, JAK/STAT, TGF-β, Wnt, Notch, and Hedgehog pathways, which may exert their protumorigenic role through crosstalk with different components of NF-κB pathways [24]. EGFR and the members of its family have been reported to activate NF-κB via PI3K/AKT signaling, mainly in estrogen receptor negative (ER^−^) breast cancers [37], while sustained activation of PI3K/AKT, MAPK, and JAK/STAT pathways in aggressive prostate cancers leads to constitutive NF-κB activity through the establishment of a cytokines-mediated autocrine loop and androgen receptor upregulation [38].

In conclusion, the aberrant NF-κB function in tumors is a guarantee for their development and progression, as well as their ability to relapse. Therefore, upregulated NF-κB detection in human malignancies is a bad sign because it is generally associated with a poor prognosis.

## 3. NF-κB in Thyroid Physiology and Pathophysiology

The role of NF-κB in thyroid physiology has not been fully elucidated, despite a number of manuscripts that have reported NF-κB activation in normal thyroid cells. Thus, it has been shown that transcription of thyroid peroxidase (TPO), a specific enzyme of differentiated thyroid follicular cells involved in the iodine organification process and one of the most common antigens linked to thyroid autoimmunity, is induced by lipopolysaccharide (LPS) through NF-κB p65/RelA phosphorylation of serine 536. Similarly, in rat thyroid FRTL-5 cells, LPS stimulated the transcription of the sodium/iodide symporter NIS, another specific gene of differentiated thyrocytes whose role is to transport bloodstream iodide into follicular cells via the cooperative activity of NF-κB and PAX8 (one of the most important regulators of NIS transcription in thyroid cells). They physically interact at the NIS mRNA upstream enhancer region that contains binding sites for both p65/RelA and PAX8. Moreover, it has also been reported that TSH receptor (TSHR) stimulation in FRTL-5 cells promotes NF-κB activation and that NF-κB signaling is needed for thyrocyte survival and the preservation of the thyroid differentiated state [20,39]. Recently, Geysels and co-workers demonstrated that TSH-induced NF-κB activation is a prerequisite for the achievement of the thyroid follicular cell differentiation program, given that p65/RelA nuclear activation promoted by TSH stimulation of thyroid cells triggers the expression of thyroid differentiation markers [40].

Since NF-κB drives the main functions of immune system cells, it is not surprising that its involvement has been proposed in thyroid autoimmunity. One of the first pieces of evidence about the interplay between NF-κB and thyroid cells has been that both IFN-α and IFN-γ induce NF-κB-mediated MHC-I expression in thyroid cells. CD40 chronic stimulation of thyroid cells, which occurs in Graves’ disease—one of the most common thyroid autoimmune disorders—leads to autoimmunity onset through the activation of both NF-κB canonical and alternative pathways [20]. In Hashimoto’s thyroiditis (HT), thyroid gland parenchyma is progressively lost and replaced by inflammatory cells that secrete chemokines, cytokines, and growth factors, the majority of which are under NF-κB regulation [17]. Thus, NF-κB may potentially contribute in different ways to thyroid autoimmunity development.

In conclusion, even though few studies are available about the definite function of NF-κB in normal thyrocytes, it is unquestionable that it participates in some of the most important activities characterizing thyroid physiology and plays an important role in many aspects of thyroid autoimmunity.

## 4. NF-κB in Thyroid Carcinomas

The first evidence of constitutive NF-κB activity in thyroid cancer cell lines was reported in 1997: in that study, the authors showed that NF-κB function was an absolute requirement for the neoplastic phenotype of thyroid cancer cells [15]. Four years later, Ludwig and co-workers demonstrated that NF-κB mediated the protumorigenic activity of *RET* oncogene in parafollicular C cells [41], while the prosurvival role of NF-κB in thyroid carcinoma cells started to be uncovered by different studies describing its ability to overcome TGF-β-, PTEN-, and radiation-induced apoptosis [42,43,44]. Subsequently, the constitutive NF-κB activity was shown for the first time in vivo through the immunohistochemical analysis of its nuclear localization in tissue sections from different human thyroid carcinoma specimens, especially from anaplastic types. In the same paper, the authors highlighted the essential role of NF-κB in the maintenance of the ATC cell transformed state and its crosstalk with the JNK pathway in the regulation of resistance to chemotherapeutic drug-mediated apoptosis [45].

**Figure 3:**
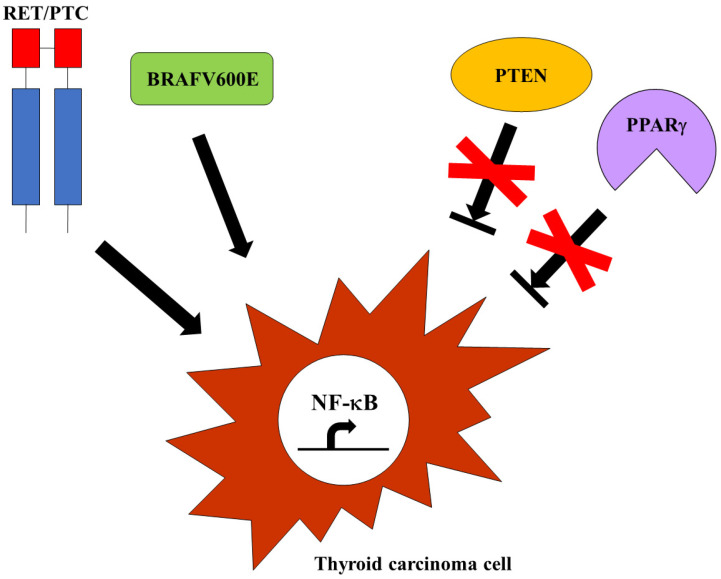
Mechanisms of NF-κB activation in thyroid cancer cells. The constitutive activation of thyroid oncogenes, such as *RET/PTC* or *BRAFV600E*, as well the suppression of tumor suppressors, such as *PTEN* or *PPARγ*, could trigger NF-κB in aggressive thyroid carcinomas.

Since the NF-κB protumorigenic function in thyroid carcinoma cells was established, a major effort has been made to unravel the molecular mechanisms by which NF-κB contributes to thyroid cancer. In particular, given the well-defined role of specific oncogenes (*RET/PTC* and *BRAFV600E*) and tumor suppressor genes (*PPARγ* and *PTEN*) in thyroid carcinogenesis, different studies aimed to investigate NF-κB involvement in the regulation of the main pathways triggering thyroid carcinoma development and progression. Thus, it has been demonstrated that PPARγ inhibition facilitates FTC onset through NF-κB-dependent cyclin D1 activation and critical apoptotic genes repression [46], and that PTEN deficiency boosted thyroid cancer progression as a consequence of NF-κB-mediated tumor cell survival enhanced by AKT activation [47]. The immunohistochemical analysis of human MTC samples harboring *RET* somatic or germline mutations showed strong nuclear staining of different members of the NF-κB family [48], corroborating the notion that mutated *RET* could be responsible for NF-κB activation in MTCs [41]. In addition, Neely and co-workers showed that RET/PTC3 was able to activate NF-κB alternative pathways in mouse embryonic fibroblasts (MEFs) and found aberrant NF-κB activation and strong NIK expression in RET/PTC3-positive PTC specimens. Therefore, they postulated that RET/PTC3 could induce the NF-κB alternative pathway in PTCs [49]. More recently, another group reported robust immunoreactivity to NIK and RelB in BRAFV600E-positive PTC samples and provided the first demonstration of the involvement of NIK and RelB, other than RelA, in the regulation of prometastatic gene expression in BRAFV600E-positive PTC cells. RNAseq data of the large PTC cohort from The Cancer Genome Atlas (TCGA) project revealed a sort of NF-κB signature, given that a set of genes with NF-κB binding sites in their promoter region was significantly enriched in *BRAF*-mutated PTCs. In particular, the upregulation of genes involved in the migration and invasion of tumor cells was strongly detected in aggressive PTCs harboring a *BRAFV600E* mutation. Interestingly, the same genes upregulated in PTCs were also found overexpressed in ATC cells, substantiating the notion that high constitutive NF-κB activity correlates with the more malignant phenotype of thyroid tumors [50]. These results confirmed and extended the findings reported by other groups that had previously pointed out the need for NF-κB signaling to promote BRAFV600E-positive PTC aggressiveness: Palona and co-workers showed in 2006 that the increased propensity of BRAFV600E PTC cells to metastasize was mainly due to the NF-κB-driven upregulation of matrix metalloproteinases [51], while Bommarito and co-workers found in 2011 that TIMP-1 bound to CD63 receptors on the surface of BRAFV600E PTC cells to promote their survival and invasiveness via AKT signaling [52]. The clinico-pathological correlation between nuclear RelA localization and BRAFV600E expression in a large cohort of highly malignant PTC cases [21], and the finding that enhanced BRAFV600E expression and constitutive NF-κB activity in tissues from PTC patients could represent a risk factor for the development of cervical lymph node metastasis [53], confirmed the pivotal role of NF-κB in mediating the aggressive behavior of BRAFV600E-positive PTCs (Figure 3).

These studies have shed more light on the main oncogenic upstream pathways able to drive NF-κB activation in thyroid cancer, especially in the highly malignant progression of thyroid neoplastic cells, and overall have clearly established that both NF-κB canonical and non-canonical signaling are involved in thyroid carcinomas. However, an important contribution to the elucidation of the molecular mechanisms allowing NF-κB to control many aspects of thyroid cancer biology arises from the identification and characterization of target genes able to mediate its protumorigenic activities in thyroid carcinoma cells.

### 4.1. NGAL

One of the most interesting NF-κB-regulated genes in thyroid neoplasias is the Neutrophil Gelatinase-Associated Lipocalin (*NGAL*), also known as Lipocalin-2 (*LCN2*). NGAL is an acute phase protein secreted by neutrophils during the inflammatory response to fight bacterial infections. In fact, NGAL is a siderophores-mediated iron binding protein that acts as a bacteriostatic agent by limiting iron availability in the microenvironment [54]. Over the years, a pivotal role of NGAL has been uncovered in different human cancers, where its expression and function correlate with tumor aggressiveness and poor outcome [55]. Its role in NF-κB-dependent thyroid cancer has been discovered and characterized for the first time in ATCs [56], where NGAL is not only strongly expressed but significantly contributes to ATC cell survival through FAS/CD95 down-regulation [57] and increases their metastatic propensity through MMP-9 enzymatic activity enhancement [58]. Moreover, in ATC cells, NGAL upregulates the expression of the different chemokines that foster leukocytes recruitment in tumor microenvironments so as to promote thyroid cancer progression [59]. Thus, NGAL upregulation in ATCs represents another way by which NF-κB could exert its protumoral activities in thyroid cancer, given the multifaceted roles covered by NGAL in ATC cells. Interestingly, *NGAL* has been identified as one of the NF-κB-dependent genes upregulated in *BRAFV600E*-mutated PTCs, whose abnormal expression correlated with advanced stages of PTC [50,60]. In addition, Tai and co-workers reported that in a cohort of pediatric PTC patients, serum NGAL levels were highly elevated and paralleled the strong NF-κB and NGAL positivity found in PTC tissues from the same patients [61]. Finally, NGAL has been proposed as a potential diagnostic marker for thyroid malignancies, since its expression is absent in normal thyroid follicular cells while it is clearly detectable in thyroid carcinomas [60,62,63,64].

### 4.2. MicroRNAs

MicroRNAs (miRNAs) play important roles in the pathogenesis of thyroid cancer: they contribute to thyroid cell transformation, increase resistance to chemotherapy, promote metastatic spread, and often represent useful tools for the diagnosis and the prognosis of thyroid carcinomas. Many signaling pathways crucial for the development and progression of thyroid cancer are affected by miRNA activity, including MAPK, PI3K, AKT, GSK-3β/β-catenin, Wnt, mTOR, and NF-κB [65]. In the majority of cases, they could aberrantly activate different members of signaling cascades, unleashing their protumorigenic power, or could be the target of their transcriptional activity that leads to miRNA abnormal expression. In thyroid carcinomas, miR-574 [66] and miR-146a [67] have been demonstrated to be under NF-κB transcriptional control and to mediate some of its protumorigenic functions. MiR-574 promotes the survival, growth, and migration of different thyroid cancer cell lines and favors tumor formation, as well as cancer-related angiogenesis in xenograft models, through the inhibition of BNIP3/AIF, which synergistically cooperate to activate proapoptotic pathways and the upregulation of MMP-2, MMP-9, and VEGF-A expression, which mediate the prometastatic behavior of thyroid carcinoma cells [66]. Similarly, miR-146a protects ATC cells from chemotherapeutic drug-induced apoptosis and supports their malignant potential. Importantly, tissue specimens from ATC patients show a perfect overlap between miR-146a overexpression and strong NF-κB nuclear staining, indicating that targeting miR-146a is one of the crucial functions of NF-κB in human ATCs [67]. The chemokine scavenger receptor D6/ACKR2 is a target of miR-146a in ATC cells: human specimens from primary ATCs show a low expression of D6/ACKR2 compared to normal thyroid tissues, suggesting an antioncogenic role for this receptor in thyroid cancer. D6/ACKR2 overexpression in ATC cells causes a dramatic loss of their ability to recruit leukocytes in vitro and in vivo, indicating that the miR-146a-mediated inhibition of D6/ACKR2 expression ensures the retention of elevated chemokine levels in tumor microenvironments to promote high rate leukocyte recruitment for thyroid cancer progression [68]. Thus, oncogenic miRNA upregulation represents a way by which NF-κB could indirectly repress the expression and the function of potential tumor suppressor genes to contribute to thyroid cancer development and progression.

### 4.3. NF-κB-Dependent Genes from Bioinformatic Analyses

A number of different genes of the NF-κB signaling pathway have been demonstrated via bioinformatic analyses to be upregulated in thyroid carcinomas with respect to normal thyroid (NT) tissues. The majority of them are mainly overexpressed in ATCs [69,70], but a significant portion is also upregulated in aggressive PTCs [50]. In particular, three genes (*PLAU, MMP-1*, and *LGALS3*)—besides *LCN2*, whose role in aggressive thyroid cancer has been discussed above—are highly sensitive to NF-κB inhibition in *BRAFV600E*-mutated PTC cells [50]. PLAU and MMP-1 primarily mediate the prometastatic activity of NF-κB in thyroid carcinomas [71,72], given that they are involved in cancer cell migration and invasion, especially in advanced thyroid malignancies [73,74,75,76]. Instead, LGALS3 is mostly used as a biomarker for thyroid cancer diagnosis, and given its participation in tumor progression, it is currently under investigation as a potential target for thyroid cancer treatment [77].

## 5. NF-κB as a Therapeutic Target in Aggressive Thyroid Carcinomas

As mentioned above, the expectations to permanently cure thyroid cancer are very high for patients with DTC, which represents the most frequent tumors affecting the thyroid gland. The same considerations cannot be made for patients with more aggressive forms of thyroid carcinomas, which often locally or distantly relapse months/years after the end of therapies (as in the case of BRAFV600E-positive PTC) or that appear difficult to cure or are even incurable after the initial diagnosis (as in the case of PDTCs and ATCs). For these patients, the most common treatment options are represented by kinase inhibitor-based therapies that target different kinases, such as RET, BRAF, RAS, MAPK, and PI3K, constitutively activated in thyroid cancer cells as a consequence of specific genetic alterations. However, high rate resistance (intrinsic or acquired) occurs during therapy, and even adopting second or third line treatments with other kinase inhibitors (alone or in combination), the prognosis remains unfavorable [1,9]. Immunotherapy, based on different approaches, including cancer vaccines, monoclonal antibodies, and immune checkpoint blockade, is the most promising therapeutic strategy for aggressive thyroid tumors expressing PD-1/PD-L1, so much so that the FDA approved in 2020 the anti-PD-1 antibody pembrolizumab for thyroid cancer treatment and a number of clinical trials have been conducted to corroborate the efficacy of spartalizumab (another anti-PD-1 antibody) for treatment of advanced or metastatic ATC [78]. Nevertheless, novel and effective approaches are needed to improve PDTC and ATC patients’ outcomes.

Since NF-κB is a main actor in aggressive thyroid carcinomas, given that it regulates many aspects of PDTC and ATC biology, novel therapeutic strategies to fight these tumors could be provided by targeting NF-κB transcriptional activity. To this end, particular interest has been given during the last few years to how one could interfere with NF-κB signaling in thyroid cancer cells. The most studied NF-κB inhibitors are listed below:

**Triptolide**—Zhu and co-workers demonstrated that triptolide, a diterpene triepoxide extracted from the Chinese herb *Tripterygium wilfordii* hook, was able to reduce ATC cell invasion through the downregulation of the NF-κB transcriptional targets cyclin D1, VEGF, and uPA, as a consequence of NF-κB inhibition [64]. Recently, it has also been reported that triptolide-mediated NF-κB inactivation also resulted in decreased proliferation and increased apoptosis of ATC cells [79].

**Bortezomib**—The proteasome inhibitor bortezomib, known for its use in multiple myeloma treatment [80], prevents IκBα degradation, thereby blocking NF-κB nuclear translocation and, consequently, its transcriptional activity [81]. It has been reported that bortezomib inhibits growth, increases apoptosis, and induces G2-M arrest in ATC cells [82], and that it sensitizes BRAFV600E-positive PTC cells to apoptosis induced by the BRAF inhibitor vemurafenib [83].

**Curcumin**—Curcumin has been reported to induce cell cycle arrest and apoptosis in thyroid cancer cells through NF-κB inhibition [84], while the simultaneous use of AZD6244, an MAPK inhibitor, and bortezomib results in a highly effective combinatorial tool to reduce the growth rate, migration, and survival of thyroid cancer cells in vitro and in vivo [85].

**Figure 4:**
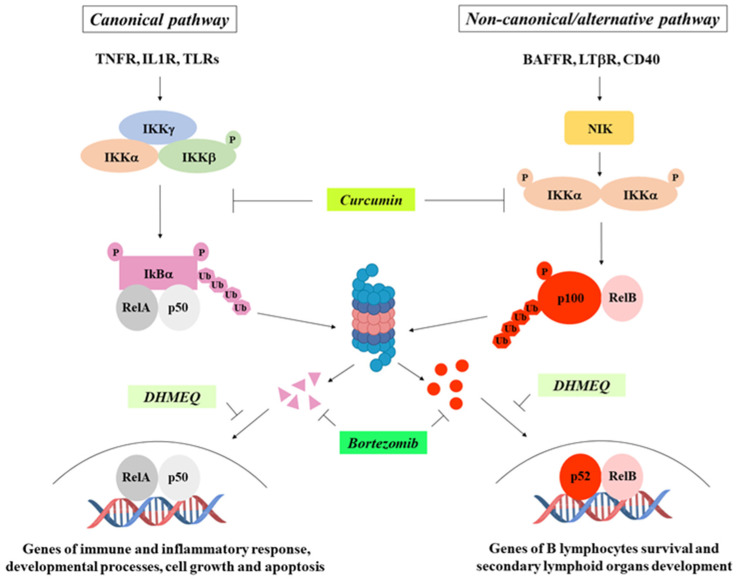
NF-κB pathways and main inhibitors. Some of the most common NF-κB inhibitors and their mechanisms of action are indicated.

**Dehydroxymethylepoxyquinomicin (DHMEQ)**—The NF-κB inhibitor DHMEQ causes apoptotic death of thyroid carcinoma cells and blocks tumor formation in nude mice [86], and reduces spheres formation in ATC cell lines without affecting their survival; and, if used in combination with the STAT3 inhibitor STA-21, the effect is synergistic and additive [23]. Since cancer stem cells play a pivotal role in the resistance to chemo- and radiotherapy, in tumor relapse and in metastases occurrence, blocking their self-renewal through NF-κB and STAT3 signaling inhibition could represent a novel therapeutic approach to improve the efficacy of chemo- and radiotherapy in the treatment of ATC patients (Figure 4).

NF-κB pharmacological inhibition also contributes to enhancing the therapeutic efficacy of ^131^I in an experimental model of DTC in vivo [87] and increases the sensitivity of PTC cells to ^131^I-induced apoptosis in vitro [88], thus providing the rationale for using NF-κB inhibitors as an adjuvant therapy for DTC patients refractory to ^131^I treatment.

Unfortunately, in some cases, the antitumoral inefficacy of NF-κB inactivation in thyroid cancer cells has been proven, as well as the failure of NF-κB inhibition to improve the therapeutic effects of antineoplastic drugs. Bauerle and co-workers have used the NF-κB inhibitors Bay-11-7082, IKK inhibitor VII, and CDDO-Me in advanced thyroid cancer cell lines and found that only one cell line shows a significant decrease in cell growth [89]. The synergistic activity of bortezomib with docetaxel or with suberoylanilide hydroxamic acid (SAHA) (a histone deacetylase (HDAC) inhibitor) is detectable in only one cell line as part of a panel including different aggressive thyroid carcinoma-derived cell lines [90]. The use of vandetanib, a RET inhibitor approved for the treatment of MTC, combined with bortezomib did not improve the efficacy of vandetanib alone in MTC patients enrolled in a phase I/II clinical trial [91].

Hence, data from the literature demonstrate that the efficacy of NF-κB inhibition is not proven in all PDTC- and ATC-derived cell lines, neither in the absence nor in the presence of drugs affecting the other main signal pathways involved in aggressive thyroid carcinomas. Nevertheless, taking NF-κB into account as a potential therapeutic target in these tumors is an attractive issue because its inactivation in PDTC and ATC cells could represent a novel weapon to associate with the gold standard therapies to make these malignancies curable. To this end, some considerations should be made: (i) NF-κB activation occurs through several pathways that share the common goal of eliciting IκBα degradation to allow NF-κB nuclear translocation. Drugs targeting upstream IκBα signal molecules cannot prevent its degradation if genetic alterations constitutively target IκBα to the proteasome independently from stimulus; (ii) the redundancy of NF-κB activating pathways raises the issue of identifying molecular targets acting as much as possible downstream of the IKK complex, which represents a sort of hub on which all NF-κB activating signals converge; (iii) since NF-κB activation could occur through a canonical and/or an alternative pathway that, as mentioned above, are often both active in aggressive thyroid cancers, targeted inhibition of only one of them could be ineffective against the other while still allowing NF-κB to carry out its functions; (iv) as in the case of other targeted therapies, drug resistance could arise after a number of treatments.

More importantly, the use of therapeutic drugs to inactivate NF-κB is currently a challenge, not only for the treatment of thyroid tumors but also for that of the majority of human cancers, so much so that the only anti-NF-κB drug approved in therapeutic regimens is bortezomib, whose employment is restricted to multiple myeloma. The main reason for the limited use of NF-κB inhibitors in cancer treatments is the risk of interfering with NF-κB physiological functions, such as immune system regulation, anti-inflammatory responses, and cellular homeostasis maintenance. Systemic NF-κB inactivation could negatively impact the response to a viral or bacterial infection as well as cause cellular stress conditions. A hypothetical way to circumvent this risk could be represented by the inhibition of NF-κB target genes that play a key role in mediating the main protumorigenic NF-κB activities in neoplastic cells, and whose suppression does not affect normal NF-κB systemic responses. In aggressive thyroid carcinomas, NGAL could be an interesting candidate gene, given its strong expression in ATC primary tumors and its involvement in many crucial functions of ATC cells. Targeting NGAL in advanced thyroid tumors is a valuable therapeutic option that could open new perspectives for the treatment of these uncurable malignancies, especially if one considers that a number of NGAL inhibitors have already been tested in different human cancers and that others are currently under investigation [92]. Besides NGAL, novel targetable genes in advanced thyroid carcinomas could emerge from the functional characterization of NF-κB-dependent genes found highly expressed in aggressive PTCs and ATCs via bioinformatic analysis [50,69,70] (Table 1).

## 6. Conclusions

The role of NF-κB in thyroid carcinomas is well established, particularly in aggressive thyroid carcinomas, but the causes of its aberrant activity in thyroid neoplastic cells are not fully understood. Many factors may contribute to the constitutive NF-κB activation in thyroid cancer: chronic extracellular stimulation of NF-κB signaling-related receptors, crosstalk with signal pathways strongly activated in thyroid carcinoma cells (MAPK, PI3K/AKT, RET/PTC, BRAF, RAS), or genetic alterations of one or more members of the NF-κB signaling cascade. Even so, it has been proposed that NF-κB could be involved in the regulation of some physiologic functions of the thyroid gland and in thyroid autoimmunity, but no evidence suggests its involvement in the onset of thyroid cell neoplastic transformation. Rather, the data indicate that NF-κB mainly participates in thyroid cancer development and progression through the upregulation of genes enhancing the proliferation, survival, and metastatic propensity of thyroid neoplastic cells. Targeting NF-κB in aggressive thyroid carcinomas represents a novel and hopeful therapeutic opportunity, but, unfortunately, it is difficult to achieve without interfering with NF-κB physiologic functions. More realistically, to overcome this hindrance, therapeutic drugs would target NF-κB-dependent genes that downstream mediate its oncogenic activity in advanced thyroid carcinomas.

## Figures and Tables

**Table 1:** NF-κB-dependent genes strongly upregulated in advanced thyroid cancer and their function in thyroid neoplastic cells.

NF-κB-Regulated Genes	Thyroid Carcinoma Expression	Impacted Activities	References
*LCN2* (Lipocalin-2)	PTC, ATC	Survival Migration Invasion	[50,56,57,58,59,60,61,62,63,64,92]
*LGALS3* (Galectin-3)	PTC, ATC	Migration Invasion	[50,77]
*PLAU* (Urokinase)	PTC, ATC	Migration Invasion	[50,70,71,73,74]
*MMP-1*	PTC, ATC	Migration Invasion	[50,72,75,76]
*miR-547*	PTC, ATC	Proliferation Survival Migration Angiogenesis	[66]
*miR-146a*	ATC	Survival Tumor progression	[67,68]
